# Ghrelin mediates exercise endurance and the feeding response post-exercise

**DOI:** 10.1016/j.molmet.2018.01.006

**Published:** 2018-01-31

**Authors:** Bharath K. Mani, Carlos M. Castorena, Sherri Osborne-Lawrence, Prasanna Vijayaraghavan, Nathan P. Metzger, Joel K. Elmquist, Jeffrey M. Zigman

**Affiliations:** 1 Division of Hypothalamic Research, Department of Internal Medicine, University of Texas Southwestern Medical Center, Dallas, TX, USA; 2 Division of Endocrinology & Metabolism, Department of Internal Medicine, University of Texas Southwestern Medical Center, Dallas, TX, USA; 3 Department of Psychiatry, University of Texas Southwestern Medical Center, Dallas, TX, USA; 4 Department of Pharmacology, University of Texas Southwestern Medical Center, Dallas, TX, USA

**Keywords:** GHSR, Ghrelin, Exercise, Treadmill, Endurance, Food intake, ACC, acetyl coA carboxylase, AMP 5′, adenosine monophosphate, AMPK, AMP-activated protein kinase, CNS, central nervous system, COX IV, cytochrome c oxidase subunit 4, G6P, glucose-6-phosphatase, GH, growth hormone, GHSR, growth hormone secretagogue receptor, HIIE, high intensity interval exercise, HNF4α, hepatocyte nuclear factor 4α, IGF-1, insulin-like growth factor-1, IGFBP-1, insulin-like growth factor binding protein-1, PC, pyruvate carboxylase, PCG1α, peroxisome proliferator-activated receptor gamma coactivator 1α, PEPCK, phosphoenolpyruvate carboxykinase, PYGL, glycogen phosphorylase, liver, RT-PCR, reverse transcriptase-polymerase chain reaction, VMH, ventromedial hypothalamus

## Abstract

**Objective:**

Exercise training has several well-established health benefits, including many related to body weight, appetite control, and blood glucose homeostasis. However, the molecular mechanisms and, in particular, the hormonal systems that mediate and integrate these beneficial effects are poorly understood. In the current study, we aimed to investigate the role of the hormone ghrelin and its receptor, the growth hormone secretagogue receptor (GHSR; ghrelin receptor), in mediating the effects of exercise on food intake and blood glucose following exercise as well as in regulating exercise endurance capacity.

**Methods:**

We used two mouse models of treadmill running to characterize the changes in plasma ghrelin with exercise. We also assessed the role of the ghrelin system to influence food intake and blood glucose after exercise, exercise endurance, and parameters potentially linked to responses to exercise. Mice lacking GHSRs (GHSR-null mice) and wild-type littermates were studied.

**Results:**

An acute bout of exercise transiently elevated plasma acyl-ghrelin. Without the action of this increased ghrelin on GHSRs (as in GHSR-null mice), high intensity interval exercise markedly reduced food intake compared to control mice. The effect of exercise to acutely raise blood glucose remained unmodified in GHSR-null mice. Exercise-induced increases in plasma ghrelin positively correlated with endurance capacity, and time to exhaustion was reduced in GHSR-null mice as compared to wild-type littermates. In an effort to mechanistically explain their reduced exercise endurance, exercised GHSR-null mice exhibited an abrogated sympathoadrenal response, lower overall insulin-like growth factor-1 levels, and altered glycogen utilization.

**Conclusions:**

Exercise transiently increases plasma ghrelin. GHSR-null mice exhibit decreased food intake following high intensity interval exercise and decreased endurance when submitted to an exercise endurance protocol. These data suggest that an intact ghrelin system limits the capacity of exercise to restrict food intake following exercise, although it enhances exercise endurance.

## Highlights

• High intensity exercise transiently increases plasma ghrelin.
• Without ghrelin action on its receptors (growth hormone secretagogue receptors), exercise markedly reduces food intake.
• An intact ghrelin system enhances exercise endurance.

## 1 Introduction

Ghrelin is a stomach-derived hormone that acts to stimulate growth hormone (GH) secretion as well as to affect various processes related to eating, body weight and blood glucose regulation [1]. In contrast to most other metabolically-acting gastrointestinal hormones, ghrelin acutely stimulates eating and also induces body weight gain upon repeated administration as a result of its orexigenic actions and its effects to reduce energy expenditure and preserve fat mass [2], [3], [4], [5], [6]. The actions of ghrelin are mediated through the growth hormone secretagogue receptor (GHSR; ghrelin receptor), which is expressed in several brain sites, the pituitary, and several peripheral organs [7], [8], [9]. GHSR activation by ghrelin requires a unique acylation of the hormone that occurs during its synthesis, although unacyl-ghrelin, which has actions *via* an as-of-yet unknown receptor, also exists in circulation [10], [11], [12]. Opposite to what might be expected based on the effects of administered ghrelin, genetic mouse models lacking ghrelin or GHSR do not demonstrate substantial differences in food intake and body weight when given free access to standard chow diet [13], [14], [15], [16], [17], [18]. As such, an intact endogenous ghrelin system does not appear to be essential to maintain normal energy homeostasis in mice during standard housing conditions – e.g. *ad libidum* access to standard chow, minimal to absent psychosocial or other types of stress, and lack of forced physical activity.

Recent studies suggest that the biological importance of endogenous ghrelin becomes accentuated during exposure to more metabolically-constrained and stressful environments. Indeed, mice lacking either ghrelin or GHSR demonstrate impaired ability to adapt metabolically and/or behaviorally to caloric restriction and psychological challenges. As such, a functional ghrelin system ensures protection from life-threatening falls in blood glucose in adult mice subjected to severe caloric restriction and in juvenile mice subjected to acute fasting [15], [16], [19], [20], [21], [22], minimizes depressive-like behaviors in mice subjected to chronic psychosocial stress, mediates the antidepressant-like and anxiolytic-like behavioral effects of caloric restriction [23], [24], and restricts body weight loss and stalls mortality associated with chronic anorexia/cachexia conditions [25]. Elevation of plasma ghrelin is a consistent feature in those challenging conditions [3], [23], [26], [27], [28], [29], suggesting that the ghrelin system is actively upregulated in those conditions as a protective measure. This upregulation of plasma ghrelin stands in contrast to the reduction in plasma ghrelin and resistance to ghrelin signaling to stimulate food intake in overly-abundant nutritional states such as obesity [30]. Therefore, an emerging notion is that the ghrelin system may serve as an essential response to metabolic and stressful challenges, minimizing perturbations to metabolic and psychological homeostasis to promote survival [12].

In this study, we aimed to study the biological significance of the ghrelin system in mice subjected to exercise as a metabolic challenge. Although the many health benefits of exercise – including weight maintenance, appetite control, improved insulin sensitivity, improved mental health, and secondary prevention of chronic diseases such as obesity, type II diabetes mellitus, cancer, and hypertension – are generally well-accepted, the molecular mechanisms that mediate and integrate these beneficial effects are poorly understood [31], [32], [33], [34], [35]. The potential role of the ghrelin system in mediating exercise capacity and the effects of exercise on food intake, body weight, and blood glucose are of particular interest given the central role of ghrelin in these processes [1], [12]. The effect of exercise on plasma ghrelin levels has been investigated in multiple human and rodent studies although the results have been inconsistent, demonstrating either a decrease, increase, or no change [36], [37], [38], [39], [40], [41], [42], [43], [44], [45], [46], [47], [48]. Notwithstanding these discrepant observations on the changes in plasma ghrelin with exercise, the impact of the ghrelin system on performance of exercise, food intake after exercise, and, more broadly, the healthy metabolic outcomes of exercise is not well-established. Here, we use two mouse models of treadmill running to characterize the changes in plasma ghrelin with exercise as well as the function of the ghrelin system to influence exercise performance, food intake, and blood glucose acutely following exercise.

## 2 Material and methods

### 2.1 Mice

All animal experiments were approved by the University of Texas Southwestern Medical Center Institutional Animal Care and Use Committee. 10–16 wk-old male GHSR-null mice [18] maintained on a C57BL/6N background (by backcrossing to C57BL/6N for many more than 10 generations over the past 10+ years) and wild-type were used in the study. The mice were generated by crossing male and female mice heterozygous for the GHSR-null allele. Mice were housed at room temperature (22–24 °C) under a 12 h dark–light cycle with free access to water and standard chow diet [2016 Teklad Global 16% protein diet (Envigo, Indianapolis, IN)], except as indicated.

### 2.2 Exercise protocols

**Figure 1:**
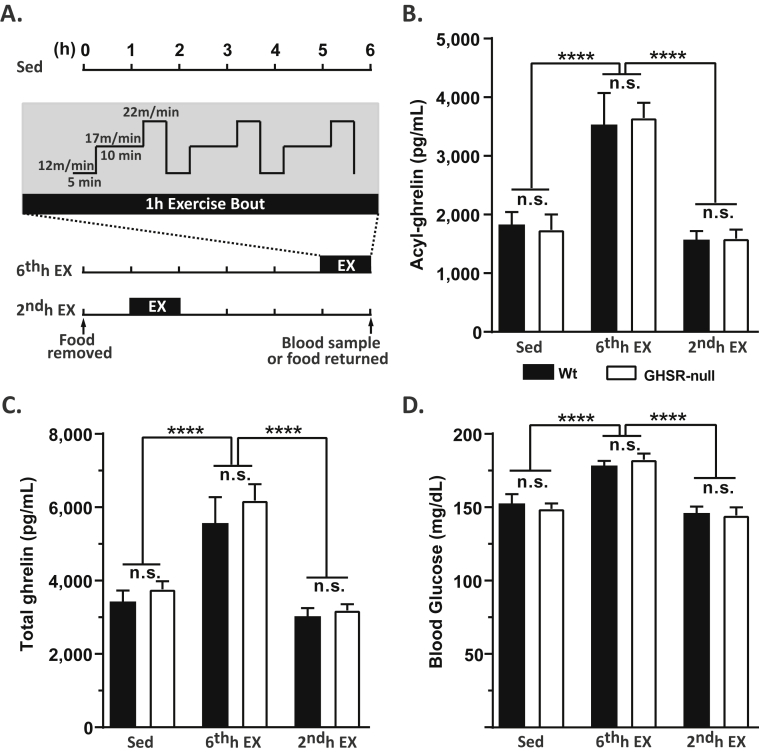
**Changes in plasma ghrelin with an acute single bout of exercise**. A. Schematic showing the timeline of the experiment. Food was removed from the mice for a total duration of 6 h and the mice were either sedentary (Sed), exercised in the 6^th^ h of food restriction (“6^th^h EX”) or exercised in the 2^nd^h of food restriction (“2^nd^h EX”). The single bout of exercise lasted for 1 h, with the speed and duration of the treadmill run as indicated in the inset and described in Materials and Methods. (B) Plasma acyl-ghrelin, (C) total ghrelin, and corresponding (D) blood glucose in wild-type (Wt) and GHSR-null littermates subjected to the 3 test conditions at the end of the 6 h experimental period. Data was analyzed by two-way ANOVA followed by Sidak *post hoc* multiple comparison test. n.s. – no significant difference, ****p < 0.001, significant difference. *n* = 12 per group. Data represented as mean ± S.E.M.

Motorized treadmills (Exer-6; Columbus Instruments, Columbus, OH) were used for exercise experiments. All mice were familiarized to the treadmills for 2 days prior to the exercise bout [Day 1: 5 min rest on the treadmill followed by running for 5 min at the speed of 8 m/min and then for 5 min at the speed of 10 m/min; Day 2: 5 min rest on the treadmill followed by running for 5 min at the speed of 10 m/min and then for 5 min at the speed of 12 m/min]. On Day 3, mice were subjected to a high intensity interval exercise (HIIE) bout (modified from Ref. [49]) to assess exercise-induced changes in plasma ghrelin, blood glucose, and food intake. Briefly, food was removed from all the mice at the start of the light cycle (7 AM) for a duration of 6 h, so as to eliminate any differences in food intake on the measured parameters (Figure 1A). Mice were rested on the treadmill for 5 min prior to performing the 1 h of exercise consisting of 3 × 20 min intervals (5 min at the speed of 12 m/min, followed by 10 min at the speed of 17 m/min, and then 5 min at the speed of 22 m/min), without rest between intervals. The 1 h exercise bout was performed either in the 6th h of food restriction (“6^th^h EX”) or the 2nd h of food restriction (“2^nd^h EX”). The two-different time points for the 1 h-duration HIIE exercise bouts were chosen so that we could more fully characterize the post-exercise physiological changes – in particular, the duration of the effects. Yet a third group of animals was kept in their home cages on Day 3 instead of being submitted to the 1 h exercise bout (sedentary; “Sed”). The mice were coaxed to continue running on the treadmill by means of an electric stimulus (0.25 mA × 163 V and 1 Hz) generated by a shock grid present at the treadmill base and by manually tapping their tails using a soft nylon bottle brush, as needed to enable all the mice to complete the exercise bout.

Exercise endurance capacity was tested by subjecting *ad libitum*-fed 10–16 wk old naïve mice to a progressive running paradigm as described previously [50], [51]. The mice were acclimatized to the treadmill for 2 days as described above. On Day 3, mice were placed on the treadmill for 5 min at rest, followed by running with a starting speed of 10 m/min for 40 min, next by running at speeds that were increased at the rate of 1 m/min every 10 min until the speed reached 13 m/min, and finally by running at speeds that were increased at the rate of 1 m/min every 5 min until exhaustion. The exhaustion time was noted as the time at which the mice stopped running and remained on the electric shock grid for 5 s, without attempting to resume running [50], [51]. Of note, for the exercise endurance capacity studies, although the electric shock grids were in place, manual coaxing with the soft nylon bottle brush was only used for the group of GHSR-null mice who were time matched to run the same duration as wild-type littermates (“GHSR-null-TM”). Sedentary controls were similarly acclimatized to the treadmill on Days 1 and 2, but on Day 3 they were instead kept in their home cages without food for a duration matching that of exercise in the exercise group.

### 2.3 Blood and tissue collection

Blood samples were collected by a quick superficial temporal vein (submandibular) bleed in the HIIE bout experiment at the end of the 6 h caloric restriction period and by decapitation in the exercise endurance capacity experiment upon exhaustion. Blood was collected from the sedentary controls using a similar procedure at the same times of day as their exercised counterparts. Blood glucose and lactate concentrations were measured immediately using the hand-held Bayer Contour and Nova Biomedical Lactate Plus™ monitoring systems, respectively. Blood also was collected into EDTA-coated vaccutainer placed on ice and immediately aliquoted into microtubes for hormone analysis. Specifically, microtubes contained p-hydroxymercuribenzoic acid (final concentration 1 mM) for ghrelin estimation, aprotinin (final concentration 250 KIU/ml) for glucagon estimation, or EDTA-glutathione solution (9% w/v EDTA and 6% w/v glutathione, pH 7.4; 2 μL per 100 μL blood) for catecholamine estimation. The blood samples were immediately centrifuged at 4 °C and 1500 *g* × 15 min. Hydrochloric acid (HCl) was added to the plasma aliquot for ghrelin estimation to achieve a final concentration of 0.1 N for stabilization of acyl-ghrelin. The processed samples were stored at −80 °C until analysis. Liver and muscle samples were harvested immediately and stored at −80 °C until used either for glycogen estimation, quantification of mRNA or protein levels, or assessment of post-translational modifications.

### 2.4 Assessment of plasma hormone levels and catecholamines

Plasma hormone concentrations were estimated using ELISA kits – Catalog #EZRGRA-90K for acyl-ghrelin, Catalog #EZRGRT-91K for total ghrelin and Catalog #EZRMGH-45K for growth hormone from Millipore-Merck (St. Charles, MO), Catalog #90080 for insulin from Crystal Chem (Downers Grove, IL), Catalog #10-1281-01 for glucagon from Mercordia AB (Sylveniusgatan, Sweden) and Catalog #EMI1001-1 for insulin-like growth factor-1 (IGF-1) from Assaypro LLC (St. Charles, MO). The total ghrelin kit estimates the concentrations of both the acyl and unacyl forms of ghrelin. The endpoint calorimetric measurements for the ELISAs were performed using a PowerWave XS Microplate spectrophotometer and KC4 junior software (BioTek Instruments, Inc. Winooski, VT). Catecholamine concentrations in the plasma were measured by High Performance Liquid Chromatography (HPLC) at the Hormone Assay and Analytical Services Core at Vanderbilt University Medical Center.

### 2.5 Assessment of liver and muscle glycogen

Glycogen content in the gastrocnemius muscle and liver collected after the endurance test were estimated using the amyloglucosidase method as described previously [52]. Briefly, the frozen samples were weighed and homogenized in ice-cold perchloric acid (0.3 N). Amyloglucosidase enzyme was then added to an aliquot of the perchloric acid extract to hydrolyze the tissue glycogen to β-d-glucose, which was then measured using the Stanbio Liqui-UV Test kit (Stanbio Laboratory, Boerne, TX) as per manufacturer's instructions.

### 2.6 Immunoblotting

Frozen gastrocnemius muscle samples were homogenized in ice cold lysis buffer composed of T-PER™ buffer (ThermoFisher, Rockford, IL), 1% (v/v) of protease inhibitor cocktail (Catalog #P8340, Sigma–Aldrich, St. Louis, MO), and phosphatase inhibitors cocktail 2 and 3 (Catalog #P5726 and P0044 from Sigma–Aldrich). The samples were homogenized for 60 s at 300 Hz using a TissueLyser II (Qiagen, Valencia, CA) and then solubilized by constant rotation for 1 h at 4 °C. Thereafter, the samples were transferred to a new 1.5 ml microcentrifuge tube and centrifuged for 10 min × 10,000 *g* at 4 °C. The supernatant was carefully pipetted into a new tube and the protein concentration was measured by Pierce BCA protein assay kit (Life Technologies, Grand Island, NY). Equal amounts of total protein (40 μg) per sample were diluted with appropriate volume of Laemmli sample buffer (2× concentrated; 4% SDS, 10% 2-mercaptoethanol, 20% glycerol, 0.004% bromophenol blue and 0.125 M Tris–HCl, pH-6.8) heated for 5 min at 95 °C, separated *via* sodium dodecyl sulfate–polyacrylamide gel electrophoresis (SDS–PAGE), and transferred to Trans-Blot Turbo™ nitrocellulose (Bio-Rad, Hercules, CA). Membranes were incubated with phospho-AMPKα (Thr172) Ab (Catalog #2531), AMPKα (F6) mouse mAb (Catalog #2793), phospho-ACC (Ser79) rabbit mAb (Catalog #11818), ACC rabbit pAb (Catalog #3662), COX IV (3E11) rabbit mAb (Catalog #4850) (Cell Signaling Technologies, Danvers, MA) and IRDye 800CW infrared dye labeled secondary antibodies (Li-Cor Bioscience, Lincoln, NB). Protein band fluorescence was quantified by using Li-Cor Odyssey Image Studio Version 4.0 (Li-Core Bioscience). Equal loading was confirmed by Coomassie staining [53].

### 2.7 Quantitative reverse-transcriptase polymerase chain reaction (RT-PCR)

Liver samples were homogenized using 5 mm stainless steel beads in a TissueLyser II bead mill (Qiagen, Germantown, MD). Total RNA was isolated using the guanidium thiocyanate-phenol-chloroform extraction method by addition of RNA STAT-60 (Amsbio, Cambridge, MA). The isolated RNA was quantified using a Nanodrop spectrophotometer (Thermo Fisher Scientific, Wilmington, DE). Total RNA (2 μg) was treated with ribonuclease-free deoxyribonuclease (Promega, Madison, WI), and complementary DNA was synthesized by reverse transcription using SuperScript III (ThermoFisher, Grand Island, NY). Primers were as used in previous publications and listed in Ref. [22]. All primers were validated by analysis of template titration and dissociation curves. Quantitative PCR was performed using an Applied Biosystems 7900HT fast real-time system (Applied Biosystems, Foster City, CA) and either SYBR green chemistry (for all the genes of interest) or TaqMan Gene Expression Master Mix (for 18S ribosomal RNA). The reaction mixture for qPCR contained the reverse-transcribed RNA, 150 nM of each primer or 1 μL 20× Assay on demand (AOD) and 5 μL of 2× iTaq Universal SYBR Green PCR master mix (Bio-Rad, Herculus, CA) or 2× TaqMan Gene Expression Master Mix (ThermoFisher). The mRNA levels were calculated using the 2^−ΔΔCt^ method. They are represented relative to the invariant control gene, 18S ribosomal RNA, and normalized to the values of the control group as indicated in the figure legends.

### 2.8 Data analysis and statistics

All data are expressed as mean ± SEM. All statistical analyses and graph preparations were performed using GraphPad Prism 7.0 (Graphpad, San Diego, CA). Student's ‘*t*’ test or two-way ANOVA followed by *post hoc* comparison tests were used to test for significant differences among test groups, as indicated in figure legends. Data with significant unequal variance assessed using Bartlett's test were log transformed prior to performing ANOVA analyses. The strength of linear relationship between two sets of variables was compared by Pearson's correlation coefficient. Outliers were detected by Grubb's test. *P*-values < 0.05 were considered statistically significant. *P*-values ≥ 0.05 and <0.1 were indicated by the actual *P* values.

## 3 Results

### 3.1 A single bout of high intensity interval exercise elevates plasma ghrelin

Plasma ghrelin is elevated in mice subjected to caloric restriction or psychological challenges. Here, we tested if challenging mice with an hour-long bout of HIIE on treadmills affects plasma ghrelin (Figure 1A). Briefly, the HIIE protocol consisted of withdrawing food for 6 h and then exercising mice on a treadmill for 1 h at the 6th h of food restriction (6^th^h EX; so as to measure physiological changes immediately after exercise), for 1 h at the 2nd h of food restriction (2^nd^h EX; so as to measure physiological changes 4 h post-exercise, by which time exercise-stimulated glucose uptake typically has subsided [49], [54], [55]), or not at all (Sed). Plasma ghrelin and blood glucose were measured at the end of the 6 h of food restriction for all the groups. Both wild-type and GHSR-null littermates, which were of equivalent body weights (data not shown), were tested, and both genotypes were able to complete the HIIE protocol. A single bout of HIIE nearly doubled the plasma acyl- and total ghrelin (Figure 1B,C), when measured immediately after exercise in the 6^th^h EX mice, when compared to the sedentary group. The plasma ghrelin levels measured 4 h after the HIIE bout in the 2^nd^h EX were comparable to those in the sedentary group, indicating that the exercise-induced elevation of ghrelin is not long-lasting. As had been shown in other conditions [24], [56], there was no difference in plasma acyl- and total ghrelin concentrations between the genotypes either in the sedentary mice or the exercised mice. Similarly, blood glucose was significantly elevated when measured immediately after exercise (in the 6^th^h EX group) in both genotypes when compared to sedentary mice, but blood glucose levels measured in mice 4 h after exercise were comparable to those of the sedentary controls (Figure 1D).

To better understand the duration of the exercise-induced ghrelin elevation, we measured plasma acyl-ghrelin at various times after the HIIE bout in the 6^th^h EX group. For these studies (and the blood glucose determinations described in the following paragraph), the 6 h of food withdrawal was extended another 2 h such that food intake would not influence the measured analytes. We used independent sets of mice for each time point (0 h, 0.5 h, and 2 h) to minimize potential influence of handling during blood collection on plasma acyl-ghrelin. Plasma acyl-ghrelin remained elevated for at least 0.5 h immediately post-exercise but significantly declined towards baseline levels by 2 h post-exercise in both genotypes (Supplementary Figure 1A). The fall in plasma acyl-ghrelin occurred more rapidly in Wt mice as compared to GHSR-nulls (Supplementary Figure 1A).

Blood glucose levels were highest immediately after the HIIE bout, significantly lower by 0.5 h post-exercise, with an even further decline noted at 2 h post-exercise (Supplementary Figure 1B). Blood glucose levels were not statistically different between the genotypes at any of the time points measured.

### 3.2 Lack of ghrelin receptors reduces food intake following exercise

**Figure 2:**
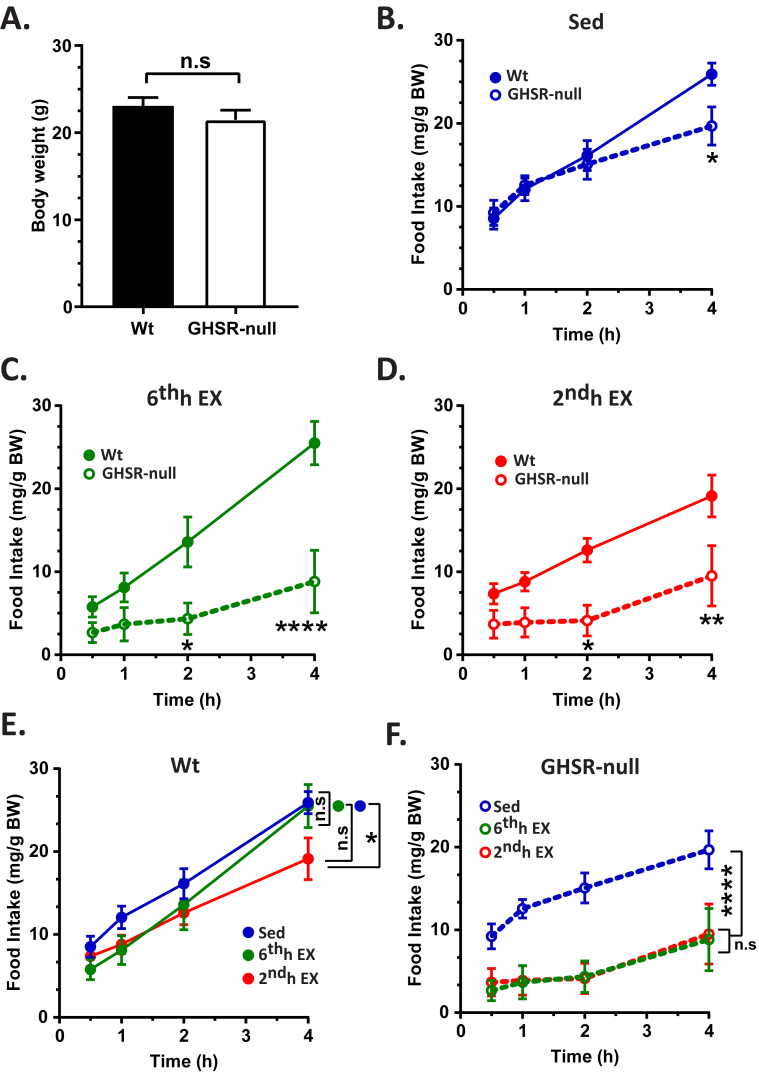
**Feeding response post-exercise in wild-type and GHSR-null mice**. A. Body weight of Wt and GHSR-null mice submitted to the HIIE protocol for studying the changes in flood intake due to lack of GHSRs. Food intake following re-introduction of standard chow to Wt or GHSR-null mice following a 6 h period of food withdrawal during which mice were either sedentary (Sed; B), exercised in the 6th h of food restriction (6^th^h EX; C), or exercised in the 2nd h of food restriction (2^nd^h EX; D). Data in B-D were analyzed by repeated measures two-way ANOVA followed by Sidak *post hoc* multiple comparison test to compare differences in food intake between genotypes at each time point. The food intake data for Wt mice represented in panels B, C, and D are combined and re-analyzed in panel E. The food intake data for GHSR-null mice represented in panels B, C, and D are combined and re-analyzed in panel F. Data in E and F were analyzed by repeated measures two-way ANOVA followed by Sidak *post hoc* multiple comparison test to compare the overall differences in food intake due to exercise. One mouse in the GHSR-null group was excluded as an outlier based on Grubb's test. n.s. – no significant difference, *p < 0.05, **p < 0.01, ****p < 0.001, significant difference. *n* = 5–6 per group. Data represented as mean ± S.E.M.

Appetite in response to exercise is highly variable and is poorly understood [35], [57]. Thus, we next assessed whether the HIIE exercise-induced elevation in circulating acyl-ghrelin acting through GHSRs affects food intake. Wt and GHSR-null mice were submitted to the HIIE protocol, just as had been described above, during the 6th or 2nd h of a 6 h food restriction or not at all. Mice were then allowed free access to standard chow beginning in the 7th h, with subsequent food intake measured after 30 min, 1 h, 2 h, and 4 h of access to food. Despite similar body weights (Figure 2A), food intake was modestly lower in sedentary GHSR-null mice when compared to sedentary Wt littermates when assessed 4 h after refeeding but was equivalent at the earlier time points (Figure 2B). Notably, exercise accentuated the difference in food intake between the genotypes in both the 6^th^h EX and 2^nd^h EX groups, with the most marked differences observed in the 6^th^h EX mice, in which the mice gained access to food immediately after the exercise bout (Figure 2C,D). To characterize food intake further, we also directly compared food intake among all the Wt groups (and all the GHSR-null groups). As compared to sedentary Wt mice, exercised Wt mice either had the same to slightly reduced food intake (Figure 2E); only with GHSR deletion was exercise able to markedly blunt food intake during the same time period (Figure 2F).

### 3.3 Lack of ghrelin receptors impairs exercise endurance capacity

**Figure 3:**
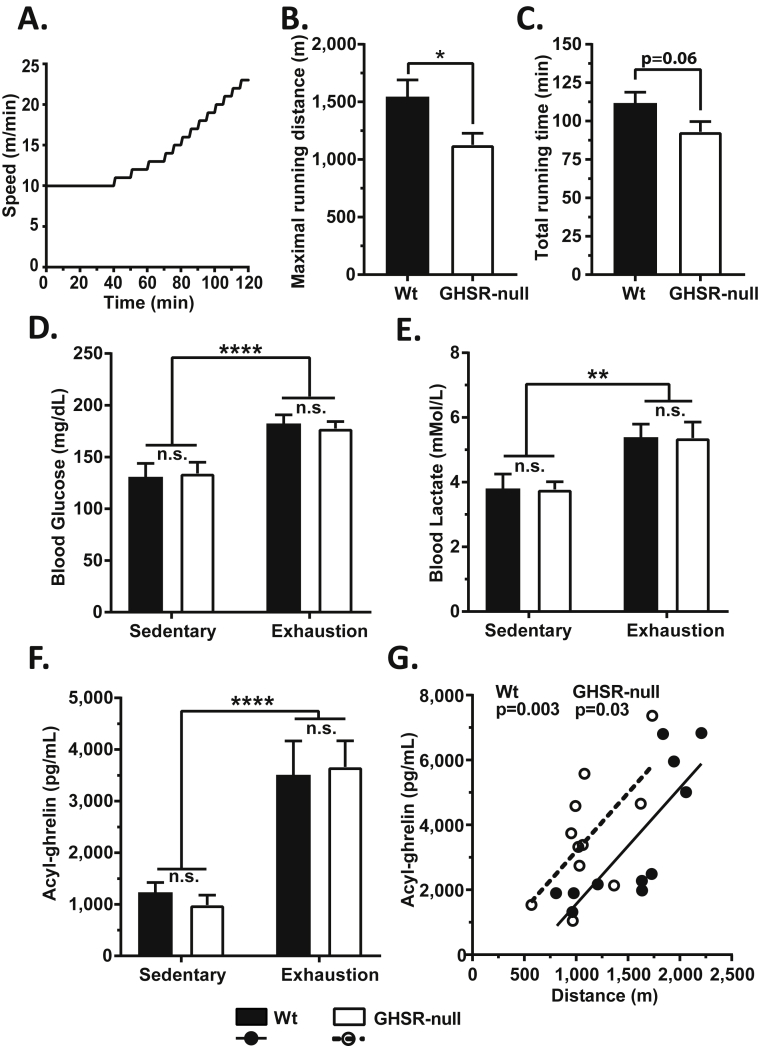
**Exercise endurance capacity and changes in blood glucose, lactate and plasma acyl-ghrelin at exercise exhaustion in wild-type and GHSR-null mice**. A. Schematic showing the exercise regimen used to test the exercise endurance capacity of Wt and GHSR-null mice. (B) Maximal distance run and the (C) duration of the exercise indicate a difference in exercise endurance between Wt and GHSR-null mice. (D) Blood glucose, (E) blood lactate, and (F) plasma acyl-ghrelin in sedentary mice vs. exercised mice at exhaustion. Data in panels B and C were analyzed by Student's unpaired “*t*” test. Data in panels D–F were analyzed by two-way ANOVA followed by Sidak *post hoc* multiple comparison test. n.s. – no significant difference, *p < 0.05, **p < 0.01, ****p < 0.001, significant difference. (G) Correlation analysis between the plasma acyl-ghrelin measured at exhaustion and the distance run until exhaustion in exercised mice. Correlation co-efficient (*r*) = 0.80 (Wt) and 0.64 (GHSR-nulls). Solid and dotted lines indicate the linear regression of correlation for the Wt and GHSR-null mice, respectively. “*p*” values indicate the significance level of the correlation. *n* = 9–12 per group. One mouse in the GHSR-null exercise group was excluded as an outlier for all the parameters, based on Grubb's test. Data represented as mean ± S.E.M.

Next, we tested if GHSR is necessary for normal exercise endurance by subjecting Wt and GHSR-null littermates to a step-wise exercise endurance protocol (Figure 3A). The GHSR-null mice exhibited significantly diminished exercise endurance capacity, reaching exhaustion after having run only about 2/3 the distance (Figure 3B) and for less time (Figure 3C) than the Wt mice. Blood glucose (Figure 3D) and lactate (Figure 3E) levels at exhaustion were significantly elevated in both the Wt and GHSR-null mice when compared to sedentary controls but did not differ between the genotypes. Exhaustion was accompanied by a roughly 3-fold elevation of plasma acyl-ghrelin in both genotypes (Figure 3F). Also, plasma acyl-ghrelin levels positively correlated with the maximal distance run until exhaustion (Figure 3G), suggesting that the extent of plasma ghrelin elevation is likely influenced by the total duration and/or intensity of exercise.

### 3.4 Lack of ghrelin receptors prevents exercise-induced increase in plasma catecholamines at exercise exhaustion

**Figure 4:**
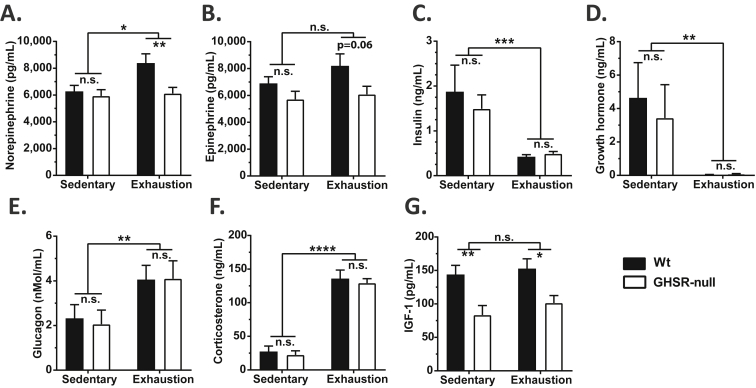
**Effect of exercise on catecholamine and hormone levels in wild-type and GHSR-null mice at exhaustion**. (A) Plasma norepinephrine, (B) epinephrine, (C) insulin, (D) growth hormone, (E) glucagon, (F) corticosterone, and (G) insulin-like growth factor-1 (IGF-1) levels in Wt and GHSR-null mice that were either sedentary or exercised until exhaustion. Data were analyzed by two-way ANOVA followed by Sidak *post hoc* multiple comparison test. n.s. – no significant difference, *p < 0.05, **p < 0.01, ***p < 0.005, ****p < 0.001, significant difference. *n* = 9–12 per group. Data represented as mean ± S.E.M.

Next, we examined potential causes for impaired endurance capacity in GHSR-null mice. Plasma norepinephrine was significantly elevated with exercise (Figure 4A). At exhaustion, plasma norepinephrine levels were significantly lower in the GHSR-null vs. Wt mice; plasma epinephrine showed a similar trend (Figure 4A,B). In both genotypes, plasma insulin and GH were significantly reduced while glucagon and corticosterone were increased at exercise exhaustion when compared to the sedentary controls, but no changes in the concentrations of these hormones were observed between the genotypes (Figure 4C–F). The insulin concentrations at exhaustion were negatively correlated with plasma acyl-ghrelin (Supplementary Figure 2). Plasma IGF-1 levels were unchanged with exercise exhaustion, but were lower in the GHSR-null mice as compared to Wt littermates (Figure 4G).

### 3.5 Effects of exercising GHSR-null mice to match the exhaustion time of Wt mice and vice versa

**Figure 5:**
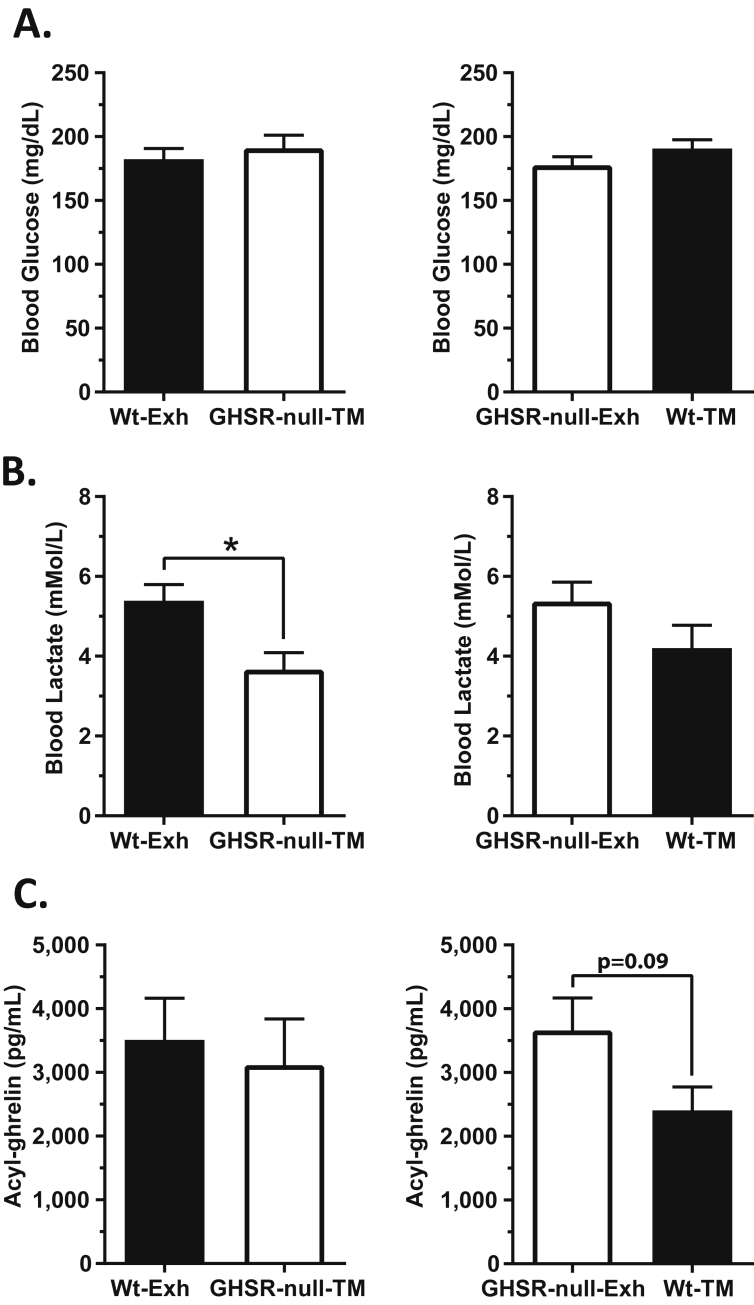
**Blood glucose, lactate, and ghrelin levels in wild-type and GHSR-null mice exercised to match the exhaustion time of the other genotype**. GHSR-null mice were exercised to match the exhaustion time of Wt mice. Similarly, Wt mice were exercised to match the exhaustion time of GHSR-null mice. (A) Blood glucose, (B) blood lactate, and (C) acyl-ghrelin in exercised mice that were time-matched (TM) to the exhaustion time (Exh) of the other genotype. Data were analyzed by Student's unpaired “*t*” test. *p < 0.05, significant difference. *n* = 7–12 per group. Data represented as mean ± S.E.M.

Next, we aimed to understand if changes in circulating concentrations of the above measured analytes help determine the impaired exercise endurance of GHSR-nulls or if they merely reflect the maximal distance run until exhaustion. We ran the GHSR-null mice to match the exhaustion time of the Wt mice by forcing them to match the average exhaustion time of Wt mice (Figure 3C) ±5 min (Wt-Exh vs. GHSR-null TM). Separately, the Wt mice were run for a duration that was time-matched to the GHSR-null mice by limiting the distance run to ±5 min of the average exhaustion time of the GHSR-null mice (GHSR-null-Exh vs. Wt-TM). No differences in blood glucose were observed in either of the time-matched groups (Figure 5A). Blood lactate concentrations in the GHSR-null mice time-matched to the Wt mice (GHSR-null-TM) were lower than those determined in Wt mice run to exhaustion (Figure 5B). The lower lactate levels in GHSR-null-TM mice vs. Wt-Exh mice suggest a relative inability of the GHSR-nulls to generate lactate or a more rapid utilization of lactate as a substrate by GHSR-nulls when forced to run to match the longer time until exhaustion exhibited by Wt mice. Plasma acyl-ghrelin was modestly lower in time-matched Wt mice as compared to the exhausted GHSR-nulls but not in time-matched GHSR-null mice as compared to exhausted Wt mice (Figure 5C). This suggests that the relative amounts of exercise-induced plasma acyl-ghrelin elevation is a function of the duration of the exercise in both Wt and GHSR-null mice, albeit when viewed together with the data presented in Figure 3F,G and Supplementary Figure 2, it seems as if the actual amounts and patterns of ghrelin secretion in response to exercise differ between GHSR-nulls and Wt mice, possibly reflecting the absence of acyl-ghrelin action in the GHSR-null group.

**Figure 6:**
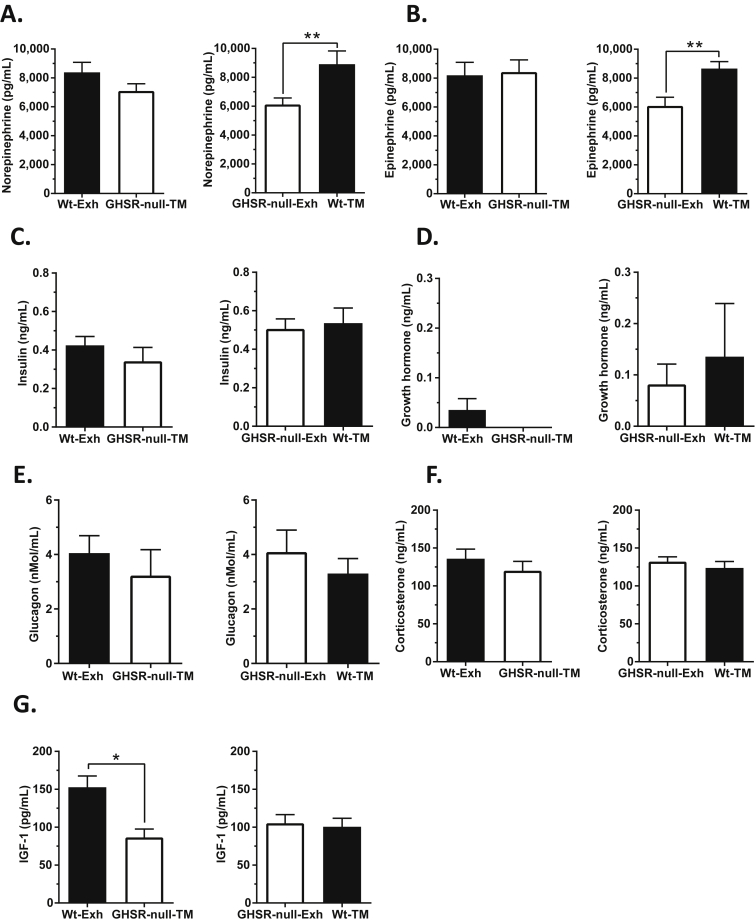
**Catecholamine and hormone levels in wild-type and GHSR-null mice exercised to match the exhaustion time of the other genotype**. Plasma (A) norepinephrine, (B) epinephrine, (C) insulin, (D) growth hormone, (E) glucagon, (F) corticosterone, and (G) IGF-1 levels in exercised mice time-matched (TM) to the exhaustion time (Exh) of the other genotype. Data were analyzed by Student's unpaired “*t*” test. *p < 0.05, **p < 0.01, significant difference. *n* = 7–12 per group. Data represented as mean ± S.E.M.

Plasma norepinephrine and epinephrine in time-matched Wt mice were significantly higher than the concentrations determined in exhausted GHSR-null mice (Figure 6A,B), similar to the results observed in exhausted Wt mice previously (Figure 4A,B). However, the catecholamine concentrations in the time-matched GHSR-nulls did not differ from those determined in exhausted Wt mice (Figure 6A,B). These observations suggest that Wt mice experience an earlier and more robust sympathoadrenal response during exercise that do GHSR-null mice, and perhaps that only with coaxed running past their natural point of exhaustion do the catecholamine levels approach the maximal level observed in Wt mice. The time-match experiments did not reveal effects on plasma concentrations of insulin, GH, glucagon, or corticosterone (Figure 6C–F). A notable difference was evident for plasma IGF-1, which was lower in time-matched GHSR-null mice as compared to the exhausted Wt mice (Figure 6G). It is not clear why the plasma IGF-1 of GHSR-null-Exh mice was not lower than that of the Wt-TM group, as had been observed in all other GHSR-null cohorts as compared to Wt mice; instead, the IGF-1 levels for the Wt-TM and GHSR-null-Exh groups were equivalent, seemingly as a result of a lower average IGF-1 level for the Wt-TM group than that observed in separate cohorts of Wt-Exh mice and sedentary Wt mice (Figure 6G, Figure 4G).

### 3.6 Lack of ghrelin receptors does not alter glycogen content, AMPK activity, ACC activity, or COX IV expression in skeletal muscle

**Figure 7:**
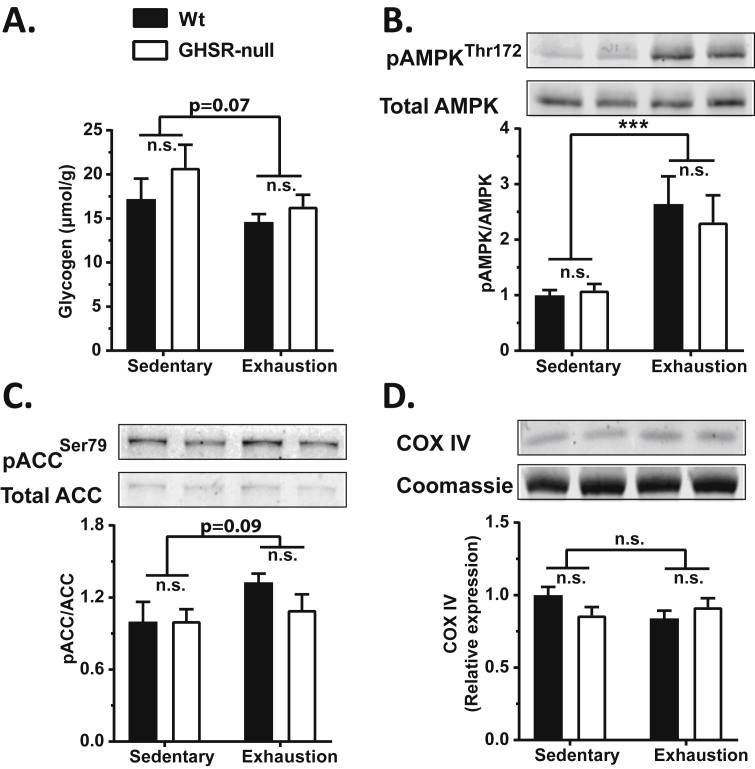
**Effect of exercise on gastrocnemius glycogen content and muscle activity markers in wild-type and GHSR-nulls**. Changes in gastrocnemius (A) glycogen content, (B) AMPK activity as measured by Thr172 phosphorylation, (C) ACC activity as measured by Ser79 phosphorylation, and (D) mitochondrial COX IV protein expression, in exercised mice. Bar graphs in B, C and D illustrate the western blot densities normalized to the average values for sedentary Wt mice. Representative western blots are shown for bar graphs B, C and D. Data were analyzed by two-way ANOVA followed by Sidak *post hoc* multiple comparison test. n.s. – no significant difference, ***p < 0.005, significant difference. *n* = 9–12 per group. Data represented as mean ± S.E.M.

We also determined if altered depletion of glycogen or deficient phosphorylation of AMPK (pAMPK) within skeletal muscle might explain the impaired exercise endurance of GHSR-nulls [58], [59], [60], [61]. Mice exercised to exhaustion showed a modest reduction in gastrocnemius glycogen content as compared to sedentary mice, with no genotype-dependent differences (Figure 7A). The reduction is glycogen in exhausted mice after exercise was accompanied by genotype-independent increases in gastrocnemius pAMPK (Figure 7B) and phosphorylation of ACC (pACC) (Figure 7C). Mitochondrial content, as assessed indirectly by measurement of mitochondrial COX IV protein levels, was neither changed by exercise nor by genotype (Figure 7D). Similarly, no changes in the abundance or phosphorylation of these proteins were observed between time-matched groups, except for modestly lower pACC in the time-matched GHSR-null mice as compared to the exhausted Wt mice (Supplementary Figure 3).

### 3.7 Lack of ghrelin receptors influences exercise induction of glucoregulatory gene expression in liver

**Figure 8:**
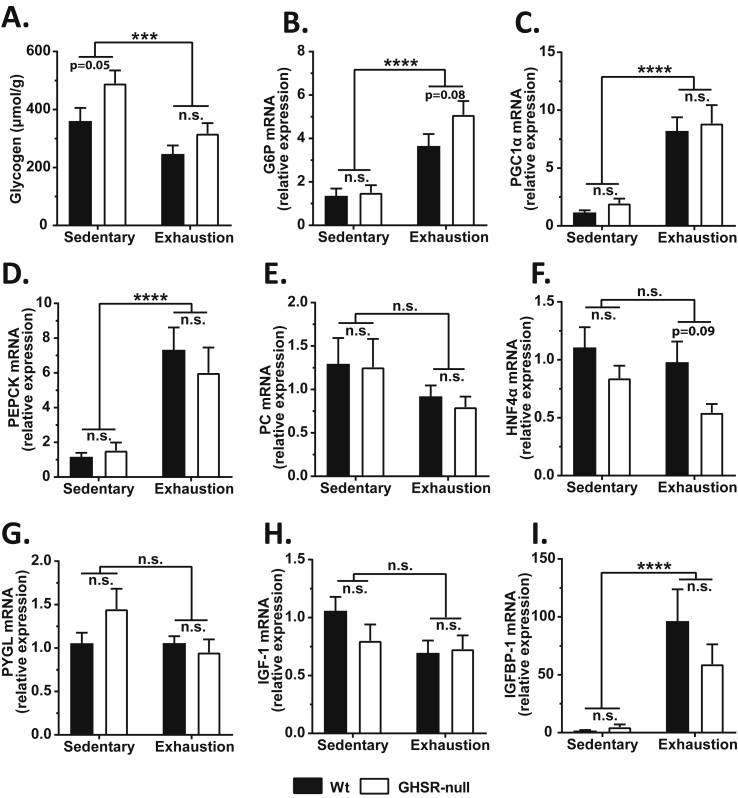
**Liver glycogen content and expression of glucoregulatory and IGF-1-related genes in wild-type and GHSR-null mice exercised until exhaustion**. The following analytes were determined for Wt and GHSR-null mice exercised until exhaustion: (A) Liver glycogen content; Messenger RNA (mRNA) expression of hepatic genes involved in gluconeogenesis: (B) glucose-6-phosphatase (G6P; *g6pc*), (C) peroxisome proliferative activated receptor, gamma coactivator 1 alpha (PGC1α; *ppargc1a*), (D) phosphoenolpyruvate carboxykinase 1 (PEPCK; *pck1*), (E) pyruvate carboxylase (PC; *pcx*), and (F) hepatic nuclear factor 4, alpha (HNF4α; *hnf4a*); mRNA expression of hepatic genes involved in glycogeneolysis: (G) liver glycogen phosphorylase (PYGL; *pygl*); mRNA expression of hepatic genes linked to IGF-1: (H) insulin-like growth factor-1 (IGF-1; *igf1*) and (I) insulin-like growth factor binding protein-1 (IGFBP-1; *igfbp1*). The mRNA expression data in panels B–I were calculated using the 2^−ΔΔCt^ method by normalizing to the Wt sedentary controls. Data were analyzed by two-way ANOVA followed by Sidak *post hoc* multiple comparison test. n.s. – no significant difference, ***p < 0.005, ****p < 0.001, significant difference. *n* = 9–12 per group. Data represented as mean ± S.E.M.

Glycogenolysis to release stored glucose and gluconeogenesis by the liver help sustain the increased energy demand during exercise. Therefore, we tested if the impaired exercise endurance of GHSR-nulls may result from differences in liver glycogen content and/or in exercise induction of the expression of hepatic genes involved in gluconeogenesis [glucose-6-phosphatase (G6P; *g6pc*), peroxisome proliferative activated receptor, gamma coactivator 1 alpha (PGC1α; *ppargc1a*), phosphoenolpyruvate carboxykinase 1 (PEPCK; *pck1*), pyruvate carboxylase (PC; *pcx*), and hepatic nuclear factor 4, alpha (HNF4α; *hnf4a*)], a gene involved in glycogenolysis [liver glycogen phosphorylase (PYGL; *pygl*)], or genes linked to IGF-1 [insulin-like growth factor-1 (IGF-1; *igf1*) and insulin-like growth factor binding protein-1 (IGFBP-1; *igfbp1*)]. Liver glycogen content was lower in exhausted mice when compared to sedentary mice (Figure 8A). While the sedentary GHSR-nulls had modestly higher liver glycogen content when compared to sedentary Wt mice (as also shown previously in GHSR-knockout mice and ghrelin-knockout mice [62]), the exhausted GHSR-nulls had similar glycogen content as that of the exhausted Wt mice (Figure 8A). Thus, even though GHSR-null mice started with a slightly higher liver glycogen content than Wt mice, and even though GHSR-null mice reached exhaustion sooner than Wt mice (Figure 3C), they seemingly utilized more liver glycogen within a shorter time. This suggests a greater rate of glycogen depletion in the GHSR-null mice during exercise. Furthermore, mice run to exhaustion had elevated expression of G6P, PCG1α, and PEPCK when compared to sedentary mice regardless of genotype (Figure 8B–D), with a slightly higher induction of G6P noted in exercised GHSR-nulls as compared to exercised Wt mice (Figure 8B). Neither exercise nor genotype affected expression of PC, HNF4α, PYGL, or IGF-1, although *post hoc* analysis did demonstrate slightly lower HNF4α mRNA in GHSR-null mice run to exhaustion as compared to Wt mice run to exhaustion (Figure 8E–H). mRNA levels of IGFBP-1, which binds to and increases the half-life of IGF-1 in circulation, was increased markedly following exercise in a genotype-independent manner (Figure 8I).

In the time-matched experiments, no differences in mRNA expression of hepatic G6P, PCG1α, or PEPCK were observed (Supplementary Figure 4A–C). However, PC mRNA expression was lower both in GHSR-null-TM mice as compared to Wt-Exh mice and in Wt-TM mice as compared to GHSR-null-Exh mice (Supplementary Figure 4D). Viewed together with the observation that the PC mRNA levels were comparable between Wt and GHSR-null mice during natural exhaustion (Figure 8E), these results suggest that PC mRNA was more rapidly induced in the GHSR-nulls than in Wt mice but that GHSR-nulls were unable to sustain those PC mRNA levels when forced to run past their natural exhaustion. HNF4α mRNA was modestly higher in Wt mice run to match the average exhaustion time of GHSR-null mice (Supplementary Figure 4E), just as had been observed in Wt mice run to exhaustion as compared to GHSR-null mice run to exhaustion (Figure 8F). Hepatic IGF-1 mRNA levels were higher in time-matched GHSR-null mice as compared to exhausted Wt mice (Supplementary Figure 4G), likely as a compensation to the lower plasma IGF-1 observed in the time-matched GHSR-null mice (Figure 6G). No differences in mRNA expression of hepatic IGFBP-1 was observed (Supplementary Figure 4H).

## 4 Discussion

In this study, we investigated the effect of exercise on plasma ghrelin as well as the impact of the ghrelin system on exercise endurance and exercise-induced changes to food intake and blood glucose. In Wt mice, a short bout of high intensity exercise was shown to transiently elevate plasma acyl-ghrelin. Without the action of this increased ghrelin on GHSRs, exercise was able to markedly reduce food intake acutely although the effect of exercise to transiently raise blood glucose remained unmodified. Furthermore, the extent of the rise in plasma ghrelin positively correlated with distance (or time) run and an intact ghrelin system was found to be required for usual exercise endurance (GHSR-null mice reached exhaustion at about 2/3 less distance). Possible factors contributing to the decreased exercise endurance of GHSR-null mice included an abrogated sympathoadrenal response as plasma norepinephrine and epinephrine were elevated in exhausted Wt mice and/or Wt mice matched to run the same time as exhausted GHSR-null mice but not in exhausted GHSR-null mice. Other potential contributors included lower overall IGF-1 levels, altered lactate metabolism, and altered hepatic glycogen utilization in GHSR-null mice. Thus, we propose that the ghrelin system normally enhances exercise endurance by stimulating the sympathoadrenal system, increasing IGF-1 levels, and/or increasing the availability of gluconeogenic substrates such as lactate to meet the energy demand of prolonged exercise.

There are several notable issues raised by our study. The first relates to the impact of exercise on plasma ghrelin levels. Plasma ghrelin increases in rodent models and humans when challenged with stressful conditions such as caloric restriction and psychosocial stress [15], [16], [20], [22], [23], [24]. Based on results from this study, we can include exercise as another challenge that increases plasma ghrelin, likely resulting at least in part from increased ghrelin secretion. Given the well-known effect of exercise to stimulate the sympathoadrenal system [63], [64], the effect of adrenergic agonists to stimulate ghrelin release [22], [65], [66], [67], and the requirement of β_1_-adrenergic receptors expressed on ghrelin cells for the elevation of plasma ghrelin during caloric restriction [22], we predict that the increase in plasma ghrelin during exercise is a direct consequence of increased adrenergic output onto ghrelin cells. The fall in insulin levels with exercise also may contribute to the increased ghrelin, as insulin is a potent negative regulator of ghrelin secretion [68].

The effect of exercise on plasma ghrelin has previously been investigated mostly in humans using exercise regimens such as treadmill running, cycling, and rowing, and also in a few rodent studies and in some other animal models. While many of these clinical studies and some preclinical studies demonstrated lower plasma ghrelin following exercise [36], [38], [39], [40], [41], [42], [43], [69], [70], higher plasma ghrelin also has been observed [37], [44], [45], [46], [71], [72], [73], as has unchanged plasma ghrelin [47], [48], [74]. The wide range of changes to plasma ghrelin could be due to the differences in the type, intensity, and duration of exercise, the metabolic and age profiles of the study subjects, the blood sample processing (which optimally is performed using a specific regimen to preserve bioactive acyl-ghrelin), and the types of ghrelin (e.g. acyl-ghrelin vs. total ghrelin) measured. In our studies, we consistently observed exercise-induced plasma acyl-ghrelin elevations, which positively correlated with the extent of exercise, although it is as yet unclear how much duration of exercise vs. distance run vs. intensity of exercise factored in to this finding. Importantly, these plasma ghrelin elevations were noted immediately at the end of the exercise bouts but were not sustained, instead falling to baseline levels by 2 h post-exercise, and thus highlighting differences in timing of the blood draw as another factor to explain some of the variability in plasma ghrelin level measurements in published human clinical trials.

Since ghrelin has the capacity to raise blood glucose [56] and as GHSR-null mice [21] along with ghrelin-knockout mice and several related models [15], [16], [19], [20] exhibit lower blood glucose levels upon caloric restriction, we had expected GHSR-null mice to exhibit lower blood glucose levels upon exercise. However, this was not observed. The lack of genotypic differences in blood glucose between Wt and GHSR-null littermates and the different trajectories of acyl-ghrelin and blood glucose (acyl-ghrelin remains elevated slightly longer than the blood glucose; Supplementary Figure 1) in the exercised mice suggest that the transient rise in blood glucose induced by the HIIE protocol is not dependent on ghrelin.

Also of note, we found that deletion of GHSR markedly reduced food intake following exercise. While there has been no consensus from clinical studies regarding the overall impact of exercise on food intake, our finding in exercised Wt mice agrees with a majority of clinical studies, which suggest that exercise either does not impact food intake or decreases short-term food intake and/or appetite [35], [75]. Some studies even have suggested that vigorous exercise reduces hunger sensations, a phenomenon referred to as “exercise-induced anorexia” [35], [76], [77]. As genetic deletion of GHSR previously has been shown to have no to only modest effects on food intake in mice with *ad lib* exposure to regular chow despite the highly potent orexigenic effects of administered ghrelin [2], [3], [4], [5], [14], [18], [78], [79], it was perhaps unexpected for the post-exercise food intake curves of GHSR-null mice to so clearly diverge from those of Wt littermates. Nonetheless, our data suggest that an intact ghrelin system is required for the usual food intake response to exercise, may last several hours post-exercise, and when blocked, may potentially amplify the efficacy of exercise to reduce post-exercise food intake.

A noteworthy topic of discussion relates to the potential processes mediating the ghrelin system's overall effect to boost exercise endurance capacity and, in particular, the decreased exercise endurance of GHSR-null mice. Differences in lactate levels and glycogen utilization were observed in GHSR-null mice as compared to Wt littermates and may be relevant to the differential exercise endurance phenotypes. Indeed, exercise endurance is thought by some to rely in part on the amount of blood lactate, which accumulates during exercise as a byproduct of the higher rate of glycolysis needed to meet skeletal muscle energy demand [80]. Importantly, GHSR-null mice coaxed to run past their natural exhaustion time to match the exhaustion time of exercised Wt mice had lower blood lactate concentrations than the Wt mice. Although GHSR deletion did not affect skeletal muscle glycogen utilization, activation of hepatic gluconeogenic genes, or skeletal muscle pAMPK, our data suggest that GHSR deletion was possibly associated with a greater rate of hepatic glycogen depletion during exercise. Furthermore, reduced levels of hepatic glycogen phosphorylase and pyruvate carboxylase mRNA were observed in GHSR-nulls forced to run as long as exhausted Wt mice. Thus, overutilization of hepatic glycogen stores and/or inefficiencies in the ability to generate sufficient amounts of gluconeogenic intermediates could reduce exercise endurance in GHSR-null mice. In fact, the inability to generate those gluconeogenic substrates has been proposed to be a key deficit that leads to severe hypoglycemia in ghrelin-knockout mice challenged with a week-long 60% caloric restriction protocol [19], [20].

Another consistent observation in our studies was the lower plasma catecholamine concentrations in the exercised GHSR-null mice, suggesting a diminished sympathoadrenal response. A proper sympathoadrenal response is essential for increasing substrate utilization during exercise [81]. Thus, the reduced sympathoadrenal response in GHSR-nulls could contribute to the reduced endurance.

GHSR deletion could also reduce exercise endurance *via* loss of ghrelin's actions to stimulate the GH axis or *via* loss of ghrelin's more direct effects on skeletal muscle strength. Not only is the well-characterized downstream effector of GH action, IGF-1, known to reduce exercise-induced skeletal muscle damage and soreness [82], [83], [84], but also IGF-1 levels were lower in GHSR-null mice. Ghrelin also may have more direct skeletal muscle-preserving and muscle strength-promoting actions during exercise. These effects presumably would mirror those of ghrelin demonstrated in several preclinical cachexia and muscle atrophy models and in cachectic patients with heart failure and chronic obstructive pulmonary disease [83], [85], [86], [87], [88], [89]. Notably, in a mouse model of chronic kidney disease, ghrelin administration was shown not only to improve muscle strength and to increase skeletal muscle mitochondria-related gene expression but also to improve exercise endurance [82], [90]. Many details regarding these latter effects of ghrelin remain unclear; interestingly, both the acyl and unacyl forms of ghrelin have been shown to mediate some of these effects [85], [91] and GHSR expression has been detected in skeletal muscle in some but not all studies [82], [91], [92], [93], [94].

The reduced exercise endurance that we observe here in GHSR-nulls contrasts with an increase in exercise endurance reported in a recent study using ghrelin-knockout mice [95]. Notable differences that might help explain these divergent results include age of mice (6–9 months-of-age and 21–24 months-of age as compared to the 2.5–4 month-old mice studied here) and intensity of exercise protocol (a high intensity endurance protocol incorporating relatively rapid increases in the treadmill speed as well as a 10° incline, leading to exhaustion before 20 min as compared to the less intense progressive endurance protocol without an incline used here, in which exhaustion was not reached until after 90 min and was associated with depletion of glycogen stores). The difference in genotype between the two studies also should not be underestimated, as loss of acyl-ghrelin-independent GHSR activity in GHSR-null mice vs. loss of desacyl-ghrelin-dependent effects in ghrelin-knockout mice potentially could differentially influence the responses to exercise of those models [12].

In the current study, we did not examine the potential impact of ghrelin action in the central nervous system (CNS) as it might relate to exercise endurance. Previously, the ventromedial hypothalamus (VMH) was identified as a key brain region required for normal exercise endurance capacity [50], [96]. The VMH is a well-known site of GHSR expression and ghrelin action [9], [97], and thus it is possible that ghrelin might act *via* the VMH or another CNS site of GHSR expression to exact its effects on exercise endurance. Neither did we assess in the current study the possible contribution of ghrelin's known actions to increase the force of cardiac contraction thus increasing stroke volume [98], erythrocyte number, and hemoglobin level [99], or vasodilation and blood flow [100] to its effects on exercise endurance – although all are possible.

## 5 Conclusions

In summary, our studies suggest that the endogenous ghrelin system is essential for exercise endurance and for the usual food intake response to exercise. From an evolutionary perspective, the ghrelin system's enhancement of exercise endurance might have evolved as a means to prolong locomotor activity thus potentially facilitating evasion of predators and food procurement especially. This concept is broadly in line with the notion of ghrelin action as an adaptation to counter metabolically and behaviorally stressful environments and to increase the likelihood of survival [12]. Previously, roles for ghrelin in engaging other types of locomotor activity have been described, including stimulation of food anticipatory behavior, spontaneous physical activity, and cocaine hyperlocomotion [79], [101], [102], [103], [104], [105], [106]. In modern society, where exercise training is one of the most effective strategies to counter the rising incidence of metabolic syndrome *via* enhanced insulin sensitivity, improved cardiovascular function, and beneficial changes in body composition [31], [107], the ghrelin system may play an important role in regulating the capacity to perform high intensity exercise while also influencing the metabolic outcome of exercise by modulating food intake. Further studies are required to understand the impact of some of the processes suggested here by which ghrelin could enhance exercise endurance, such as an enhanced sympathoadrenal response, elevated plasma IGF-1 levels, altered lactate metabolism and glycogen utilization, as well as direct effects on skeletal muscle contractility, CNS action, and cardiac function. Given the marked decrease in food intake in GHSR-null mice observed here following the HIIE exercise bout, while also keeping in mind their reduced exercise endurance, we believe it would be meritorious to investigate the pros and cons of using GHSR antagonists as an adjunctive therapy to the commonly-recommended treatment plan consisting of dietary intervention plus exercise that forms the mainstay of most current weight loss regimens.

## Appendix A Supplementary data

Supplemental Figure 1: Blood glucose and plasma ghrelin trajectories post-exercise. (A) Plasma acyl-ghrelin and blood glucose (B) in Wt and GHSR-null mice measured at 0 h (immediately after exercise), 0.5 h, and 2 h after a single 1 h bout of HIIE. Blood sampling for acyl-ghrelin and glucose estimation at various time points were performed in independent sets of mice. The mice were food restricted for 8 h and exercised in the 6^th^ h of food restriction. Data was analyzed by two-way ANOVA followed by Sidak *post hoc* multiple comparison test. n.s. – no significant difference, ***p* < 0.01, ****p* < 0.005, *****p* < 0.001, significant difference. *n* = 8–12 per group. Data represented as mean ± S.E.M.

Supplemental Figure 2: Plasma acyl-ghrelin negatively correlates with insulin during exercise exhaustion. Correlation analysis between plasma acyl-ghrelin and plasma insulin measured at exercise exhaustion. Correlation coefficient (*r*) = −0.51 (Wt) and −0.73 (GHSR-nulls). Solid and dotted lines indicate the linear regression of correlation for the Wt and GHSR-nulls, respectively. “*p*” values indicate the significance level of the correlation. *n* = 9–12 per group. Data represented as mean ± S.E.M.

Supplemental Figure 3: Muscle activity markers in wild-type and GHSR-null mice exercised to match the exhaustion time of the other genotype. (A) AMPK activity as measured by Thr172 phosphorylation, (B) ACC activity as measured by Ser79 phosphorylation, and (C) mitochondrial COX IV protein expression in Wt and GHSR-null mice exercised to match the exhaustion time of the other genotype. The data were derived by normalizing the Western blot densities to the average values for the exhausted Wt or GHSR-null mice. Data were analyzed by Student's unpaired “*t*” test. *n* = 7–12 per group. Data represented as mean ± S.E.M.

Supplemental Figure 4: Expression of glucoregulatory genes in wild-type and GHSR-null mice exercised to match the exhaustion time of the other genotype. (A) Glucose-6-phosphatase (G6P; *g6pc*), (B) peroxisome proliferative activated receptor, gamma coactivator 1 alpha (PGC1α; *ppargc1a*), (C) phosphoenolpyruvate carboxykinase 1 (PEPCK; *pck1*), (D) pyruvate carboxylase (PC; *pcx*), (E) hepatic nuclear factor 4, alpha (HNFα; *hnf4a*), (F) liver glycogen phosphorylase (PYGL; *Pyg*l), (G) insulin-like growth factor-1 (IGF-1; *Igf1*) and (H) insulin-like growth factor binding protein-1 (IGFBP-1; *Igfbp1*) in Wt and GHSR-null mice exercised to match the exhaustion time of the other genotype. The mRNA expression data were calculated using the 2^−ΔΔCt^ method by normalizing the data of the time match-group to that of the exhaustion group (Wt-Exh or GHSR-null-Exh) in each graph. Data were analyzed by Student's unpaired “*t*” test. **p* < 0.05, ***p* < 0.01, ****p* < 0.005, significant difference. *n* = 7–12 per group. Data represented as mean ± S.E.M.
