# Supplementary figures and images for: Ghrelin mediates exercise endurance and the feeding response post-exercise
Source: Mol Metab. 2018 Jan 31;9:114–30. doi: 10.1016/j.molmet.2018.01.006 (PMC5870098; doi:10.1016/j.molmet.2018.01.006)

# Western blots of gastrocnemius ACC phosphorylation

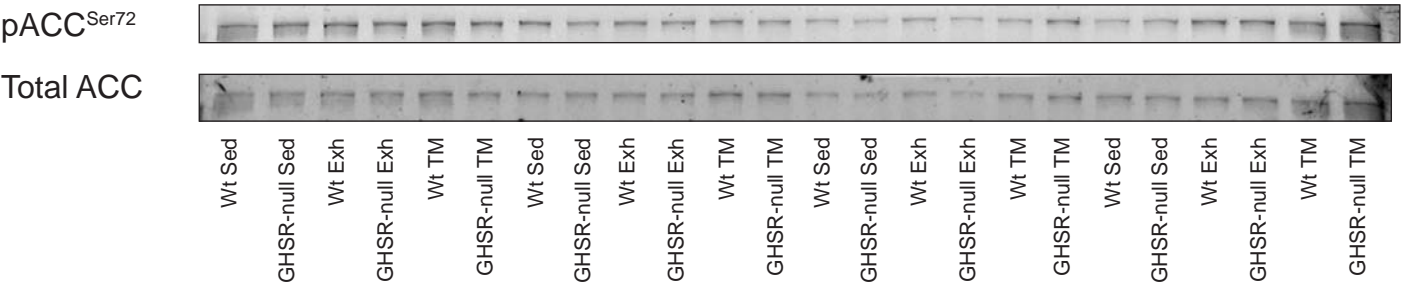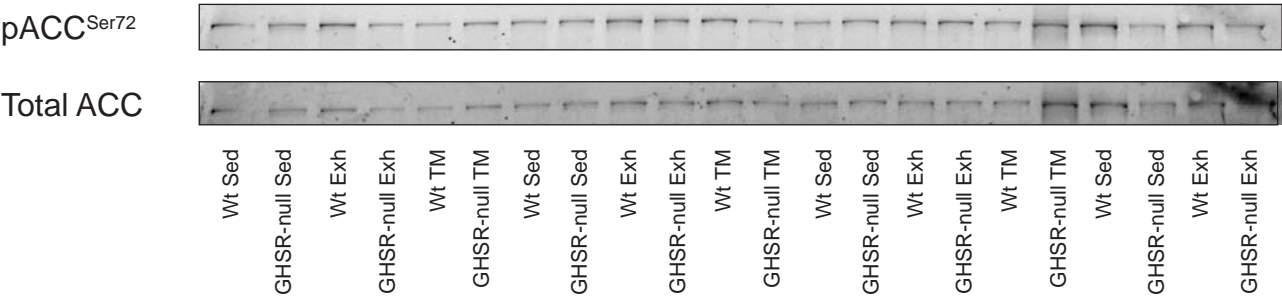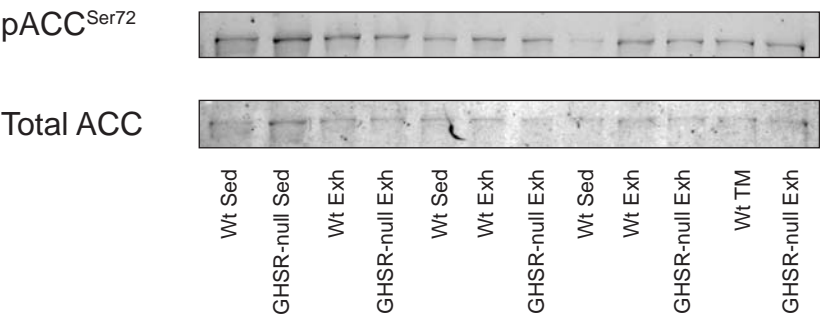

Supplement: Supplementary file 1 [file mmc1.pdf]

# Western blots of gastrocnemius AMPK phosphorylation

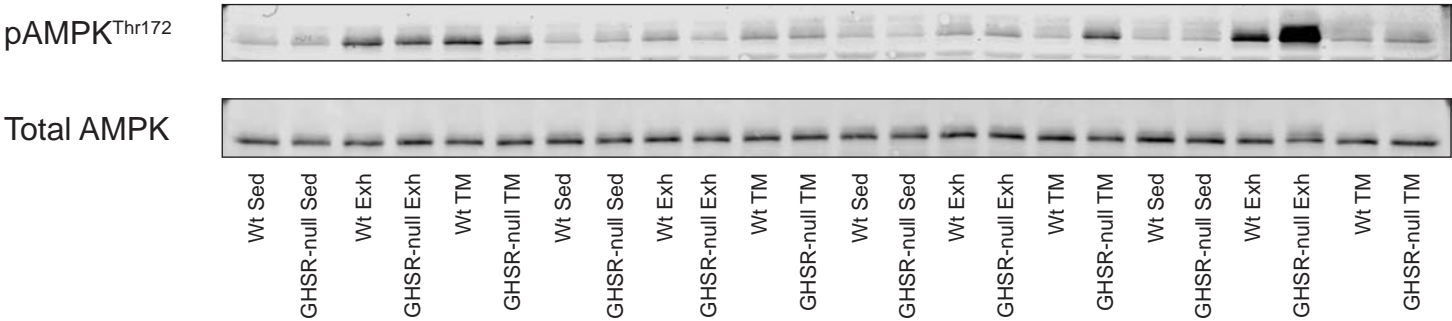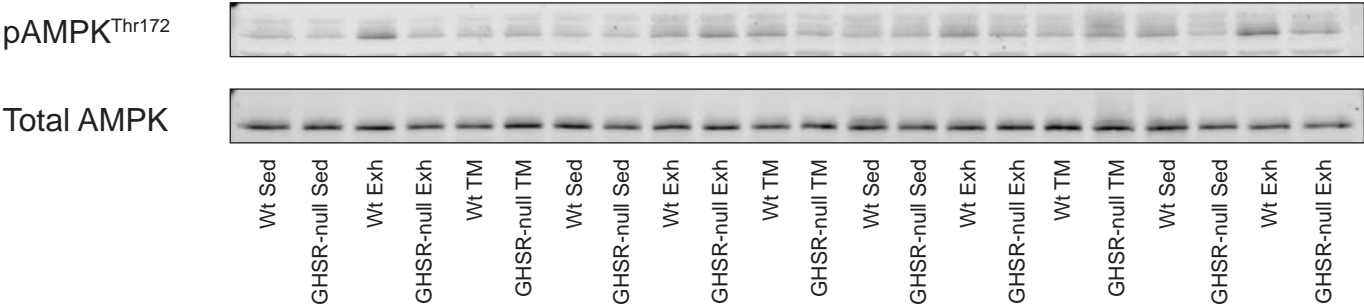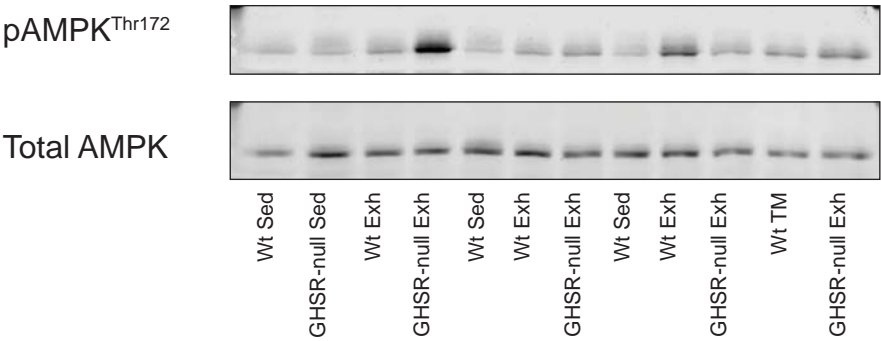

Supplement: Supplementary file 2 [file mmc2.pdf]

# Western blots of gastrocnemius COX IV expression

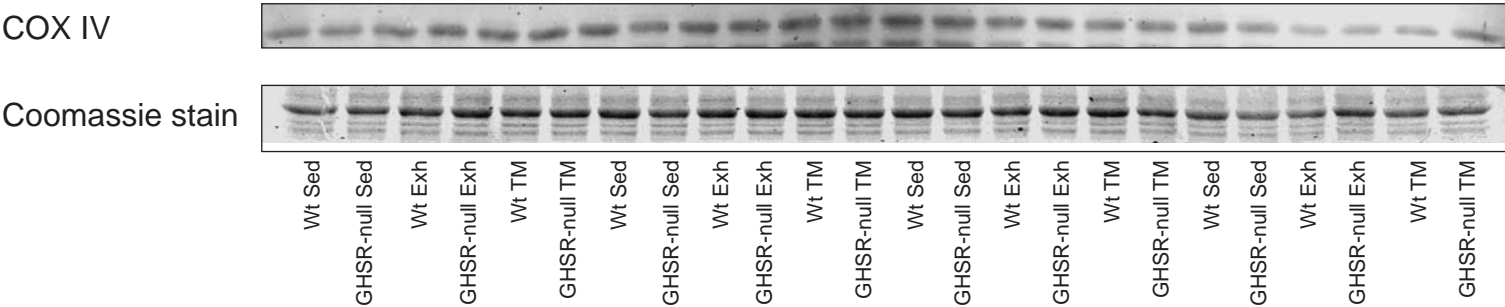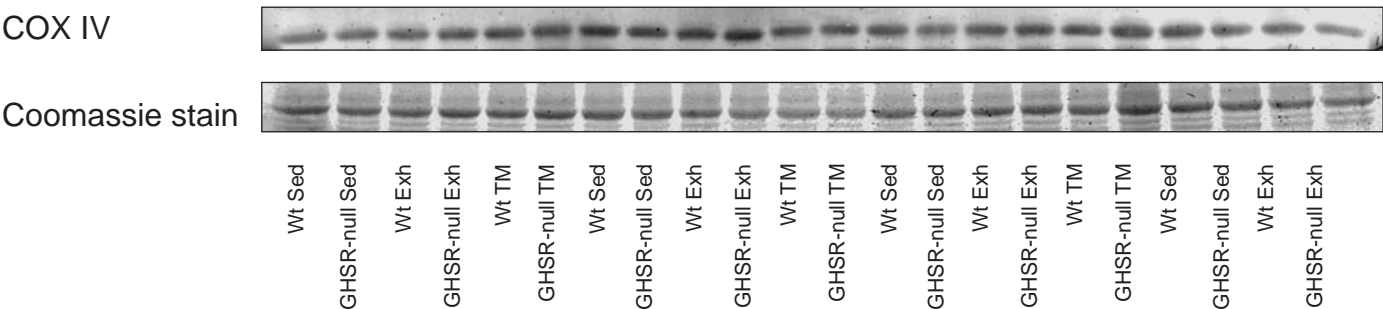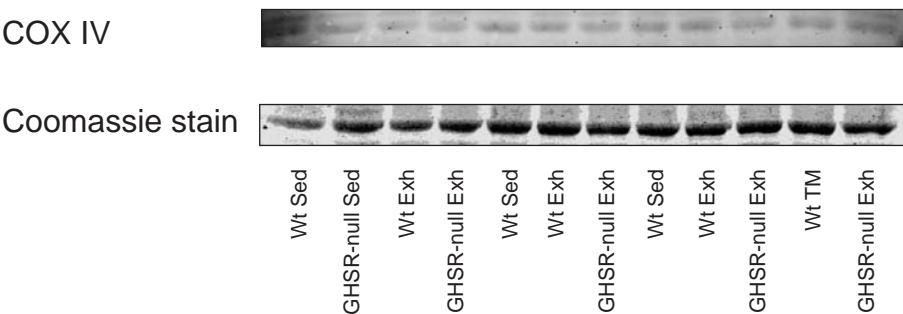

Supplement: Supplementary file 3 [file mmc3.pdf]

## Supplemental Figure 1

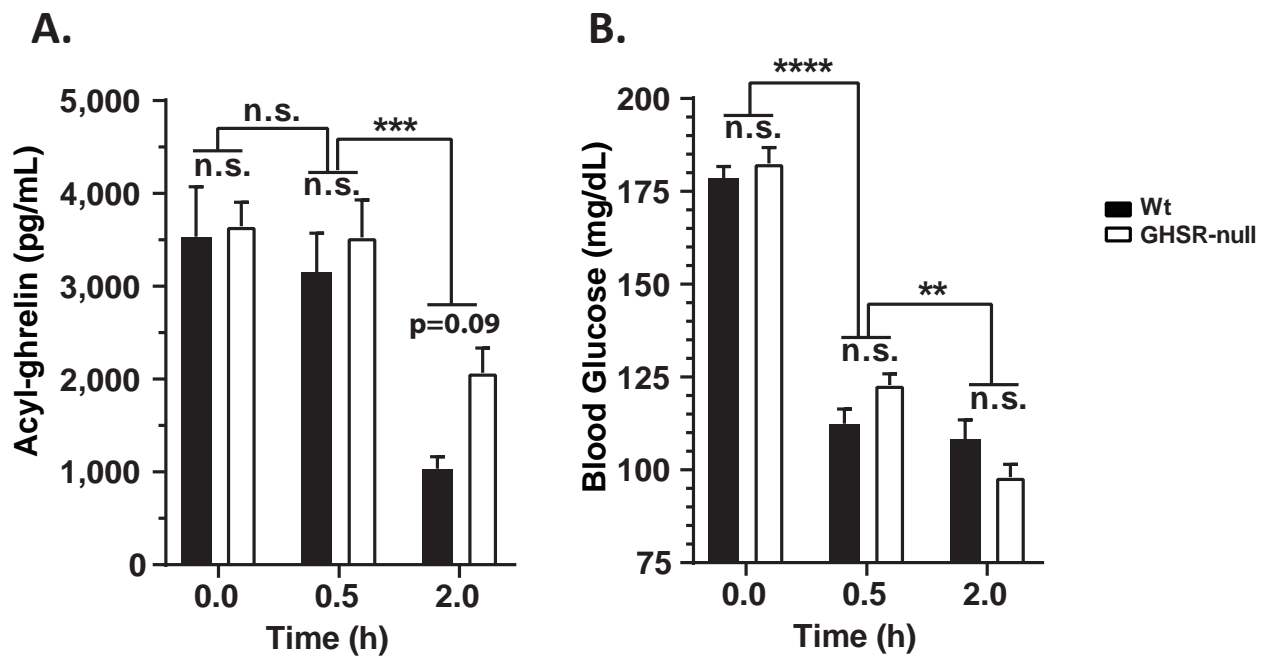

Supplement: Supplementary file 4 — Supplemental Figure 1: Blood glucose and plasma ghrelin trajectories post-exercise. (A) Plasma acyl-ghrelin and blood glucose (B) in Wt and GHSR-null mice measured at 0 h (immediately after exercise), 0.5 h, and 2 h after a single 1 h bout of HIIE. Blood sampling for acyl-ghrelin and glucose estimation at various time points were performed in independent sets of mice. The mice were food restricted for 8 h and exercised in the 6th h of food restriction. Data was analyzed by two-way ANOVA followed by Sidak post hoc multiple comparison test. n.s. – no significant difference, **p < 0.01, ***p < 0.005, ****p < 0.001, significant difference. n = 8–12 per group. Data represented as mean ± S.E.M. [file mmc4.pdf]

Supplemental Figure 2

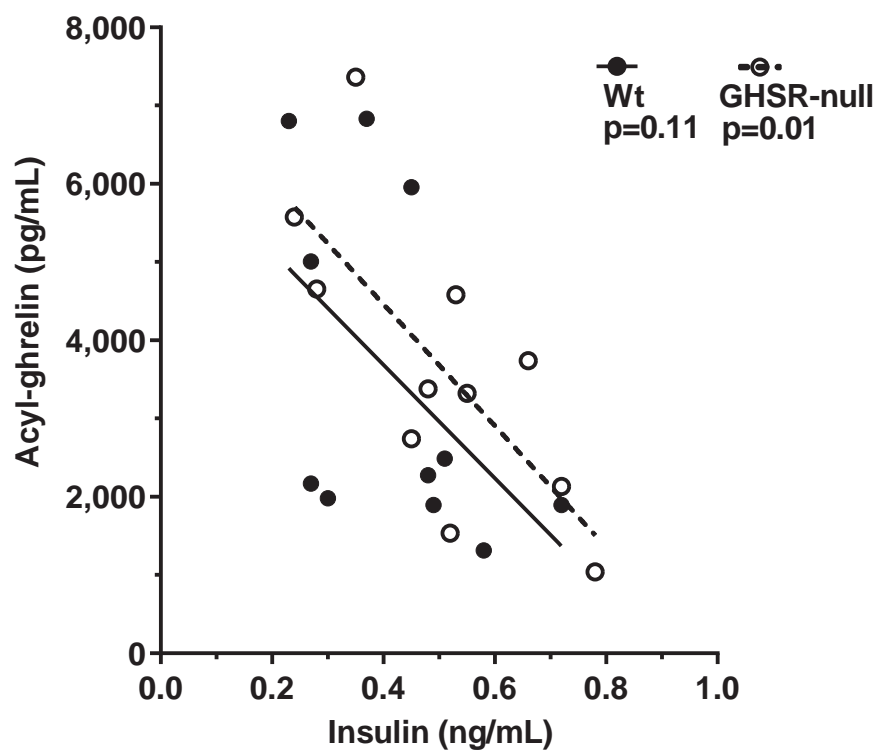

Supplement: Supplementary file 5 — Supplemental Figure 2: Plasma acyl-ghrelin negatively correlates with insulin during exercise exhaustion. Correlation analysis between plasma acyl-ghrelin and plasma insulin measured at exercise exhaustion. Correlation coefficient (r) = −0.51 (Wt) and −0.73 (GHSR-nulls). Solid and dotted lines indicate the linear regression of correlation for the Wt and GHSR-nulls, respectively. “p” values indicate the significance level of the correlation. n = 9–12 per group. Data represented as mean ± S.E.M. [file mmc5.pdf]

## Supplemental Figure 3

**A.**

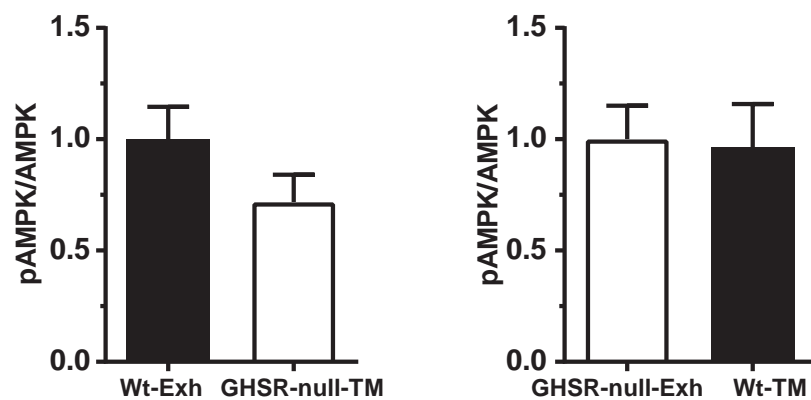

**B.**

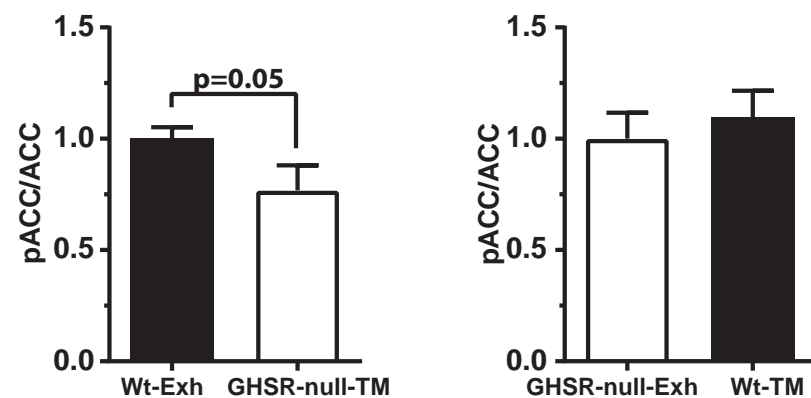

**C.**

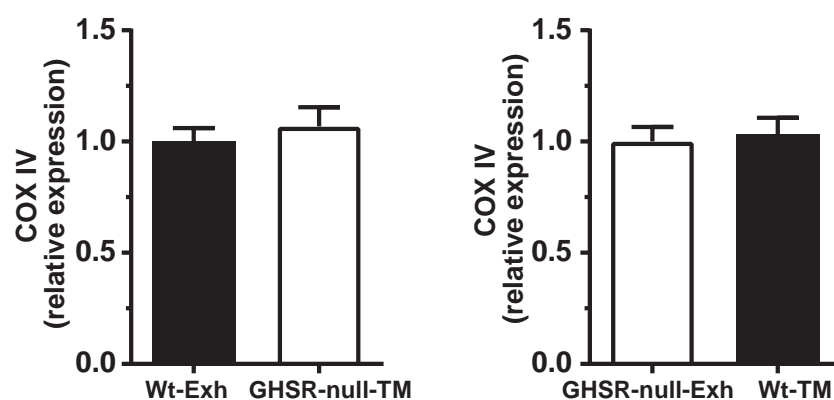

Supplement: Supplementary file 6 — Supplemental Figure 3: Muscle activity markers in wild-type and GHSR-null mice exercised to match the exhaustion time of the other genotype. (A) AMPK activity as measured by Thr172 phosphorylation, (B) ACC activity as measured by Ser79 phosphorylation, and (C) mitochondrial COX IV protein expression in Wt and GHSR-null mice exercised to match the exhaustion time of the other genotype. The data were derived by normalizing the Western blot densities to the average values for the exhausted Wt or GHSR-null mice. Data were analyzed by Student's unpaired “t” test. n = 7–12 per group. Data represented as mean ± S.E.M. [file mmc6.pdf]

## Supplemental Figure 4

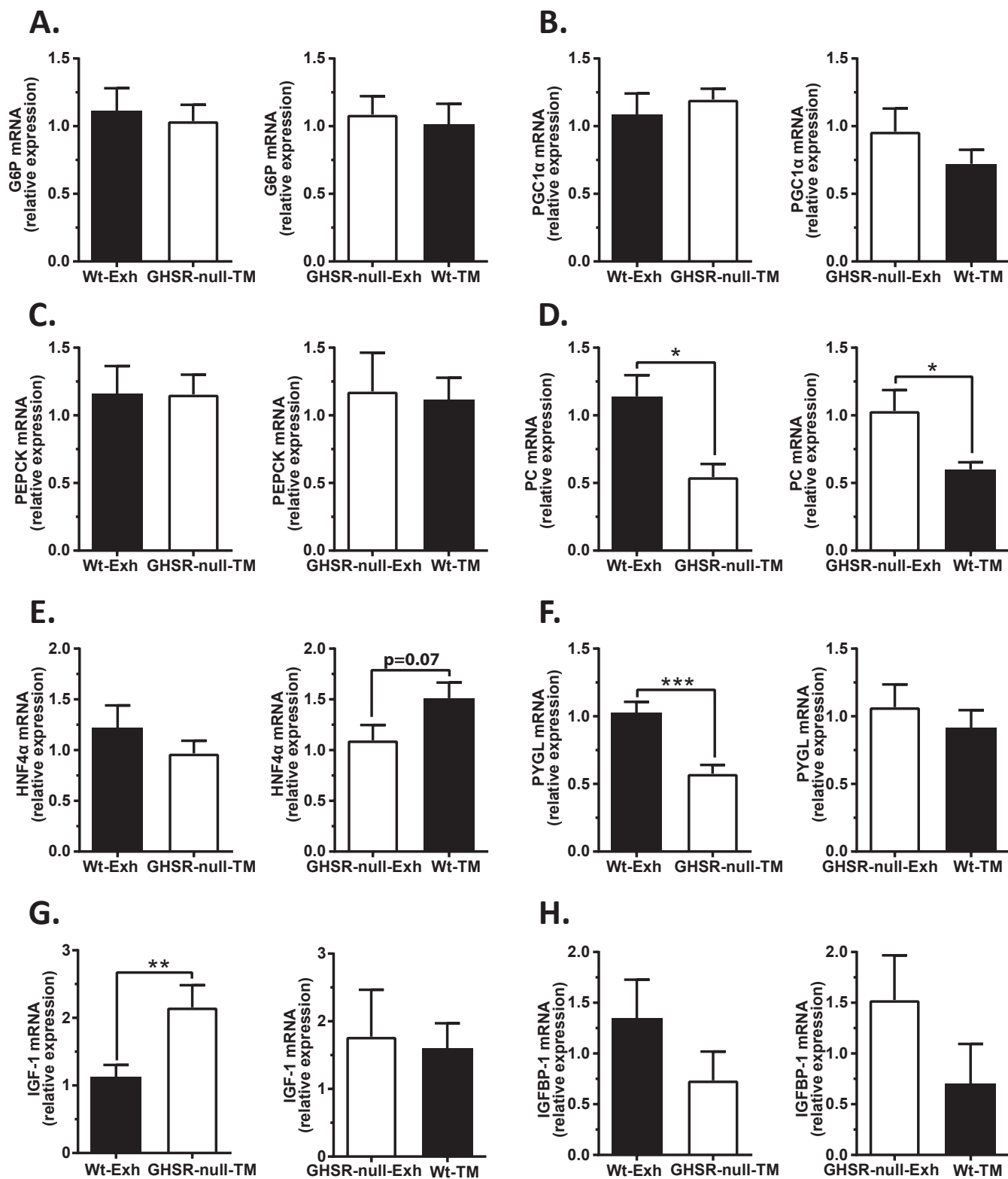

Supplement: Supplementary file 7 — Supplemental Figure 4: Expression of glucoregulatory genes in wild-type and GHSR-null mice exercised to match the exhaustion time of the other genotype. (A) Glucose-6-phosphatase (G6P; g6pc), (B) peroxisome proliferative activated receptor, gamma coactivator 1 alpha (PGC1α; ppargc1a), (C) phosphoenolpyruvate carboxykinase 1 (PEPCK; pck1), (D) pyruvate carboxylase (PC; pcx), (E) hepatic nuclear factor 4, alpha (HNFα; hnf4a), (F) liver glycogen phosphorylase (PYGL; Pygl), (G) insulin-like growth factor-1 (IGF-1; Igf1) and (H) insulin-like growth factor binding protein-1 (IGFBP-1; Igfbp1) in Wt and GHSR-null mice exercised to match the exhaustion time of the other genotype. The mRNA expression data were calculated using the 2−ΔΔCt method by normalizing the data of the time match-group to that of the exhaustion group (Wt-Exh or GHSR-null-Exh) in each graph. Data were analyzed by Student's unpaired “t” test. *p < 0.05, **p < 0.01, ***p < 0.005, significant difference. n = 7–12 per group. Data represented as mean ± S.E.M. [file mmc7.pdf]
